# Fat-signal suppression in breast diffusion-weighted imaging: the Good, the Bad, and the Ugly

**DOI:** 10.1007/s00330-024-10973-4

**Published:** 2024-08-07

**Authors:** Denis Le Bihan, Mami Iima, Savannah C. Partridge

**Affiliations:** 1 NeuroSpin, Joliot Institute, Commissariat à l’Energie Atomique (CEA), Paris-Saclay University, Bât 145, CEA-Saclay Center, 91191 Gif-sur-Yvette, France; 2 Human Brain Research Center, Kyoto University Graduate School of Medicine, Kyoto, Japan; 3 National Institute for Physiological Sciences, Okazaki, Japan; 4 Department of Fundamental Development for Advanced Low Invasive Diagnostic Imaging, Nagoya University Graduate School of Medicine, Nagoya, Japan; 5 Department of Diagnostic Imaging and Nuclear Medicine, Kyoto University Graduate School of Medicine, Kyoto, Japan; 6 Department of Radiology, University of Washington School of Medicine, Seattle, WA USA; 7 Breast Imaging, Fred Hutchinson Cancer Center, Seattle, WA USA

**Keywords:** Fat suppression, Diffusion-weighted magnetic resonance imaging, Breast imaging, Signal to noise ratio, Quantitative imaging

## Abstract

**Objectives:**

Fat-signal suppression is essential for breast diffusion magnetic resonance imaging (or diffusion-weighted MRI, DWI) as the very low diffusion coefficient of fat tends to decrease absolute diffusion coefficient (ADC) values. Among several methods, the STIR (short-tau inversion recovery) method is a popular approach, but signal suppression/attenuation is not specific to fat contrary to other methods such as SPAIR (spectral adiabatic (or attenuated) inversion recovery). This article focuses on those two techniques to illustrate the importance of appropriate fat suppression in breast DWI, briefly presenting the pros and cons of both approaches.

**Methods and results:**

We show here through simulation and data acquired in a dedicated breast DWI phantom made of vials with water and various concentrations of polyvinylpyrrolidone (PVP) how ADC values obtained with STIR DWI may be biased toward tissue components with the longest T1 values: ADC values obtained with STIR fat suppression may be over/underestimated depending on the T1 and ADC profile within tissues. This bias is also illustrated in two clinical examples.

**Conclusion:**

Fat-specific methods should be preferred over STIR for fat-signal suppression in breast DWI, such as SPAIR which also provides a higher sensitivity than STIR for lesion detection. One should remain aware, however, that efficient fat-signal suppression with SPAIR requires good B0 shimming to avoid ADC underestimation from residual fat contamination.

**Clinical relevance statement:**

The spectral adiabatic (or attenuated) inversion recovery (SPAIR) method should be preferred over short-tau inversion recovery (STIR) for fat suppression in breast DWI.

**Key Points:**

- *Fat-signal suppression is essential for breast DWI; the SPAIR method is recommended.*
- *Short-tau inversion recovery (STIR) is not specific to fat; as a result, SNR is decreased and ADC values may be over- or underestimated.*
- *The STIR fat-suppression method must not be used after the injection of gadolinium-based contrast agents*.

## Introduction

In 2020 we published an article in *European Radiology* with the title “*Six DWI questions you always wanted to know but were afraid to ask: relevance for breast diffusion MRI*” [1]. We thought that this was a timely contribution, as the tremendous growth of diffusion MRI (or diffusion-weighted imaging, DWI) since its introduction in the mid-1980s and its widespread utilization, especially in oncology, required some critical attention to standardization and quality control. We then focused on six questions taking breast DWI for context, as breast DWI is increasingly used in clinical practice. We showed especially that noise effects, of which authors and readers may be genuinely unaware, could lead to incorrect data interpretations or conclusions. Here, we investigate another issue that was not addressed in our article: Fat. Contamination of DWI signals by fat may also have some pernicious effects, in particular in the breast, which often has a high-fat content. In the present article, our goal is to show how fat signal affects DWI, qualitatively and quantitatively, and how fat effects can be corrected. Indeed, several methods exist for DWI fat suppression (see refs. [2–5] for a review). The most popular approaches by far for fat-signal suppression in breast DWI are the STIR (short-tau inversion recovery) and SPAIR (spectral adiabatic (or attenuated) inversion recovery) methods because they are efficient and clinically easy to implement [2–4]. Hence, this article focuses on those two techniques to illustrate the importance of appropriate fat suppression in breast DWI, briefly presenting the pros and cons of both approaches and concluding that SPAIR is preferable to the STIR approach, especially for quantitative breast DWI.

### The importance of fat-signal suppression in breast DWI

Fat contributes to MRI signals via its hydrogen nuclei, which have a slightly different MRI resonant frequency (chemical shift) than water hydrogen nuclei (the difference is, on average, around 3.5 parts per million, i.e., about 220 Hz at 1.5 T and 440 Hz at 3 T). Their frequencies are so close that, without special care, fat and water signals are mixed up within each voxel signal. The problem is that the diffusion coefficient of fat hydrogen nuclei is more than 10 times lower than that of water. Hence, signals from voxels containing fat exhibit artifactually lower absolute diffusion coefficient (ADC) values than in the absence of fat. This ADC underestimation depends on the fat content (proton-density fat fraction) (ESM Fig. 1) and the nature of the fat. This pitfall has long been recognized in the liver, especially in the context of hepatic steatosis [6], but is also of concern for breast DWI. Low ADC values are most often associated with malignancies, which has led the European Society of Breast Imaging (EUSOBI) International Working Group on Breast DWI [7] to suggest ADC threshold values may be used to classify breast lesions. Hence, fat contamination may lead to false-positive cases.

Another drawback of the presence of fat in breast DWI is the presence of severe chemical shift-related ghost artifacts, as DWI is usually performed using an echoplanar imaging (EPI) readout which is very sensitive to slight differences in resonance frequencies [8]. In the phase-encoding direction, the bandwidth-per-pixel is very small (around a few Hz/pixel) to allow the acquisition of all k-space lines in a single shot and mitigate the occurrence of motion artifacts. A fat/water chemical shift of 220 Hz may thus shift the fat component of each voxel in the phase-encoding direction by several millimeters depending on the acquisition parameters, resulting in a fat ghost image overlapping the water image.

Hence, it is clear that fat-signal suppression is mandatory for breast DWI [7, 9]. Several methods exist: chemical shift-based methods (such as the Dixon [10] and CHESS [11] techniques) selectively target (eliminate) the fat signals based on their resonant frequency, as it is slightly different than water. Unfortunately, the spectrum of fat hydrogen nuclei (e.g., triglycerides, etc.) is broad, unlike water, resulting in many distinct frequencies, with peaks overlapping each other, especially at low field strength. For this reason, frequency selective pulses, even in the absence of B0 inhomogeneities (good shimming) will not excite all fat hydrogen nuclei.

By contrast, other techniques rely on the short T1 of fat, such as the STIR method, but this approach also has limitations, which are the object of this article. The most serious pitfall of the STIR method is that it is not at all specific to fat: all tissue components with a T1 value close to that of fat will be erased from the DWI signal. Other tissue components will also exhibit a signal decrease, depending on their T1 value (ESM Fig. 2). Overall, this means that DWI signals will be attenuated, leading to a general loss in signal:noise ratio (SNR) in already low SNR DWI images. Because of the drawbacks of both approaches, hybrid methods have been introduced, such as the spectral presaturation with inversion recovery (SPIR) and the SPAIR technique, which are independent of tissue T1 values. To highlight our presentation of those issues we have run simulations and acquired DWI data with the STIR and SPAIR techniques on a dedicated breast phantom.

## Materials and methods

*Simulations* were conducted to mimic signals of breast lesion components (T1 = (700–2000 ms), ADC = (0.7–2.0 × 10^-3 ^mm²/s) within fibroglandular tissue (FGT) (T1 = 1300 ms, ADC = 1.6 × 10^-3 ^mm²/s). Signals were calculated at *b* = 0 and 800 s/mm² as:

$$\qquad\qquad {{S}}\left({{b}}\right) = \left[{{{{\rm{FS}}}}}.{{{{\rm{g}}}}}\, \left({{{{\rm{T}}}}}1,{{{{\rm{T}}}}}2\right).\exp \left(-{{b}}.{{{{\rm{ADC}}}}}\right)\right]$$

FS = | (1–2exp(− TI/T1)| for STIR (TI = 200 ms), FS = 1 for SPAIR, and *g* (T1, T2) is the T1/T2 weighting for a spin-echo sequence. The ADC corresponds to the value obtained at *b* = 800 s/mm² (in tissues this ADC would intrinsically include both Gaussian and non-Gaussian diffusion effects [12]). To mimic a heterogeneous, multi-component lesion, signals *S*_i_(0) and *S*_i_(800) from several components, i, were added:

$$\qquad\qquad\qquad\quad {{S}}\left({{b}}\right) = \mathop{\Sigma }_{{{{{\rm{i}}}}}}\, \left[{{{\,f}}}_{{{{{\rm{i}}}}}}.{{{S}}}_{{{{{\rm{i}}}}}}\left({{b}}\right)\right]$$

where *f*_i_ is the component fraction. The multi-component ADC was obtained from the composite signals as ln(*S*(*b* = 0)/*S*(*b* = 800))/800.

### Phantom study

**Fig. 1:**
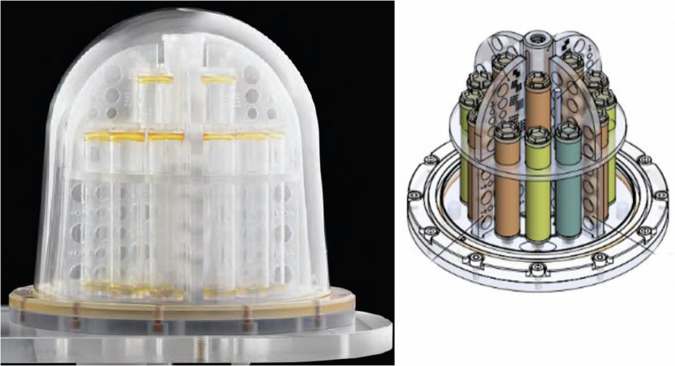
Phantom used to illustrate the effects of STIR and SPAIR on breast DWI fat suppression. Breast DWI phantom (CaliberMRI, Inc., [13]) containing vials of water, FGT, and fat mimic (T1_fgt_ = 1219 ms T1_fat_ = 296 ms), and different concentrations of polyvinylpyrrolidone (PVP), ranging from 10% to 40%

DWI data were acquired at 3T (Achieva, Philips Healthcare) on a breast DWI phantom unit (CaliberMRI Inc.) [13]) consisting of vials containing water, FGT and fat mimics (with T1_fgt_ = 1388 ms and T1_fat_ = 319 ms at 20 °C), and different concentrations of polyvinylpyrrolidone (PVP), ranging from 10% to 40% to achieve different diffusivities (Fig. 1). Single-shot EPI was performed using *b*-values of 0 and 800 s/mm^2^ (TR = 8657 ms, TE = 77 ms, 1 average, resolution: 2.1 mm × 2.1 mm). Fat suppression was performed using SPAIR and STIR with TI values of 200 ms and 300 ms. All the other protocol parameters were set according to the ACRIN 6698 study and QIBA DWI Profile [14]. The ADC and signal:noise ratio at *b* = 800 s/mm² was estimated for each vial. ROIs were drawn within each vial.

### Clinical cases

To illustrate the inaccuracy of ADC obtained using STIR (over or underestimation), DWI data from two example cases were selected from an existing imaging research trial patient cohort (one patient with normal breast with a fluidic component and another patient with invasive ductal carcinoma) acquired under IRB approval with a waiver for informed consent. MR images were obtained at 3 T (Prisma; Siemens Healthineers) using an 18-channel dedicated breast coil. DWI EPI images were acquired with *b* = 0 and 800 s/mm², TR = 7020 ms, TE = 58 ms, voxel size 2.0 × 2.0 × 3.0 mm, and two averages. 3D ROIs were drawn manually to cover the lesions.

## Results

### Qualitative effects: SNR and CNR

**Fig. 2:**
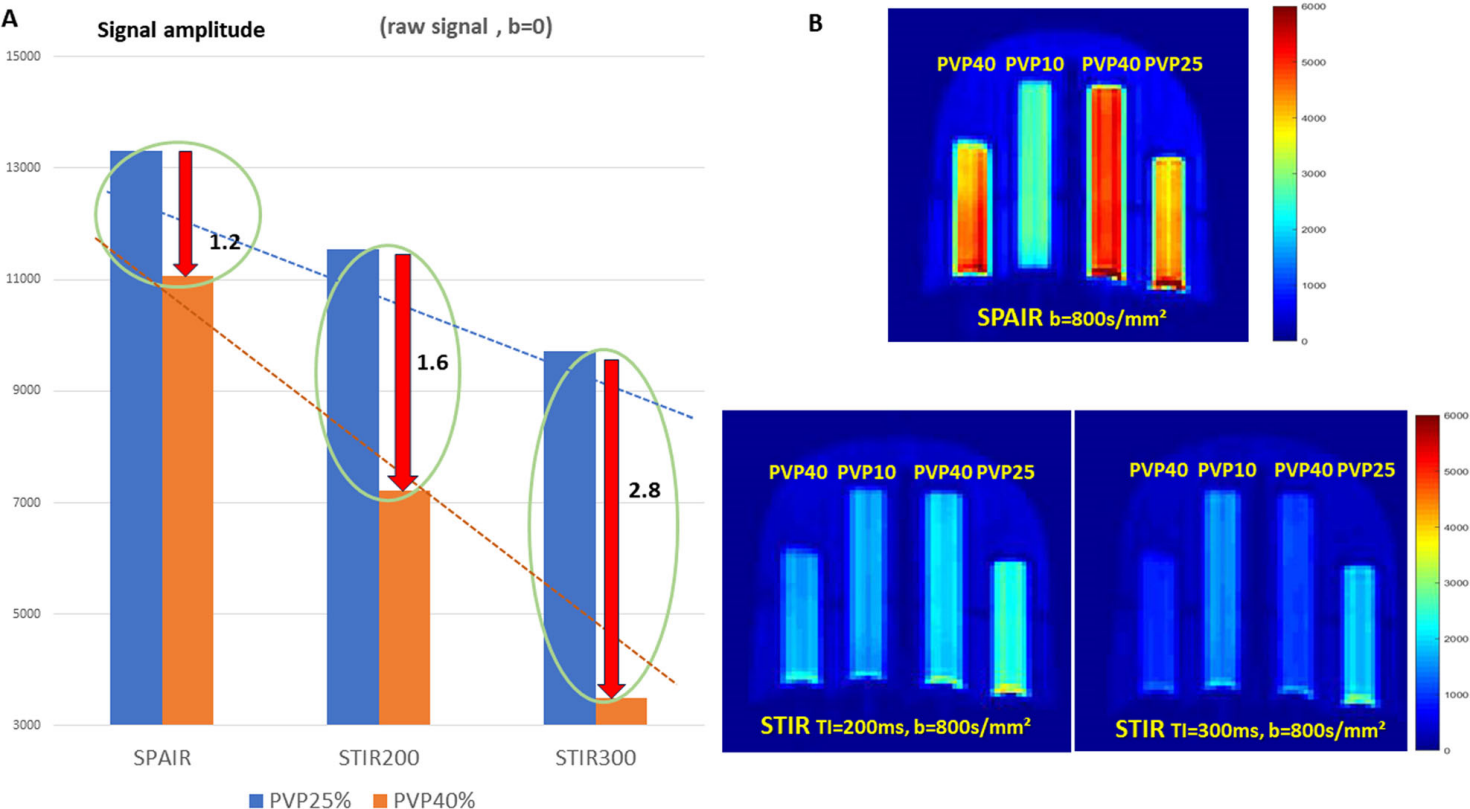
**A** (left): signal level at *b* = 0 s/mm²; **B** (right): SPAIR and STIR DWI images at *b* = 800 s/mm². **A** The residual signal with STIR is deeply reduced compared to SPAIR according to the TI/T1 ratio of the vials (the absolute values of raw signal are used), especially with TI = 300 ms. **B** SNR maps (represented at the same color scale): At *b* = 800 s/mm² the contrast between vials is flattened out with STIR because of the combined effect of T1 (reduced signal for short T1 components) and diffusion (reduced signal for high diffusion). Vial T1s are given in Fig. 4. Note that in a malignant breast lesion (long T1 and reduced diffusion and long T1) the aspect will be opposite, with the lesion appearing as brighter

As mentioned above the STIR method is not at all specific to fat: all tissue components exhibit a signal decrease, depending on their T1 value, as seen in the phantom vials. While the signal decreases when T1 is shorter (e.g., for PVP 40% compared to PVP 25%), the signal drop is much larger for STIR, especially with the TI value increases, as required when B0 increases (Fig. 2A). As this drop in DWI signals will be larger for tissues with short T1s, contrast might be flattened out in STIR DWI images as diffusion effects (higher signal for low diffusion) may be offset by T1 effects (lower signal for short T1) in such tissues (Fig. 2B).

**Fig. 3:**
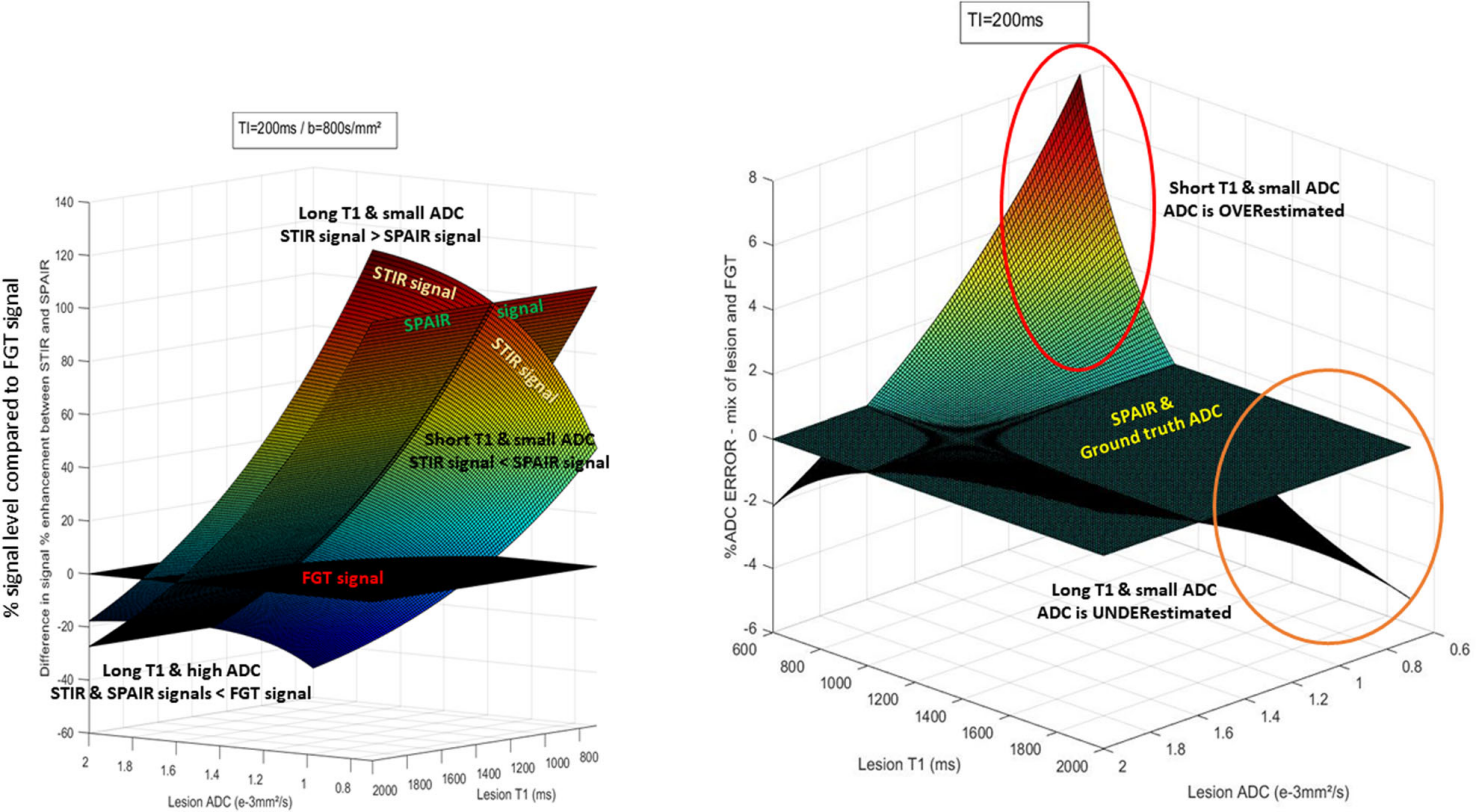
Left: STIR and SPAIR signals relative to FGT (*b* = 800 s/mm²); right: ADC error for STIR compared to ground truth and SPAIR ADC. Left: lesions with low ADC appear brighter (more visible) over background FGT with STIR if T1 is long, but brighter with SPAIR if T1 is short. Lesions with high ADC will appear darker than FGT. Right: the resulting ADC of a heterogeneous lesion (or ROI) with a low ADC is overestimated for lesions with short T1 and underestimated for lesions with long T1 (the simulated lesion (or ROI) was made of two components, one with fixed values of T1 = 1000 ms and ADC = 1.6 10^-3 ^mm²/s)

However, in malignant breast lesions which usually have long T1 and low ADC values the overall effect would be opposite (lesions appearing as brighter due to combined T1 and diffusion effects). Hence, qualitatively at least, this pitfall may not be such bad news, as diffusion-weighted images acquired at high *b*-values will increase the contrast between malignant lesions which have both longer T1 values and lower ADC values than the background FGT (Fig. 3). This trick, combined with the MIP technique, is exploited by the diffusion-weighted imaging with background suppression method (DWIBS) [15] to improve the detection of lesions from the background.

Still, this apparently enhanced contrast will highly depend on the components present in lesions, depending on their relative T1 and ADC values (ESM Fig. 3): At *b* = 800 s/mm² due to the combination of T1 and diffusion effects, STIR signals relative to FGT signal appeared higher than SPAIR for tissues with long T1 and low ADC values, but lower for tissues with short T1 and high ADC values (Fig. 3 left).

### Quantitative effects: ADC

**Fig. 4:**
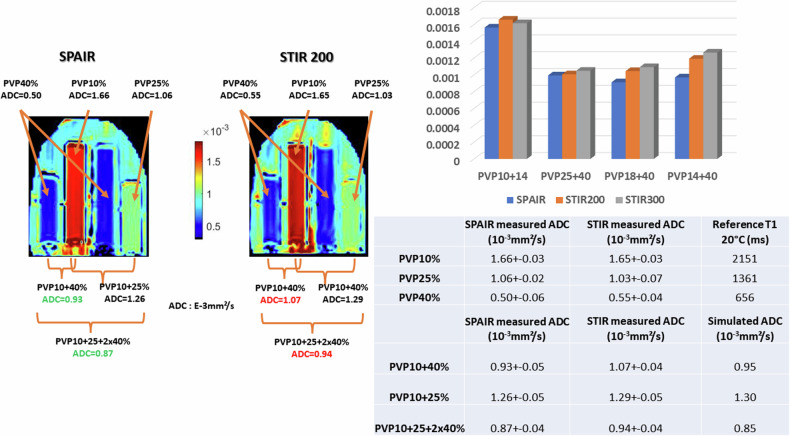
Phantom ADC maps obtained using SPAIR and STIR (TI = 200 ms). Left: in homogeneous vials, ADC values (10^-3^mm²/s) obtained with SPAIR and STIR are comparable. However, when vial contents are mixed, ADC values become over- or underestimated with STIR, depending on the respective T1 and ADC values of mixed vials. In tissues where low ADC values would be associated with long T1s, the STIR ADC values would be underestimated. Right, bottom: The ADC values obtained in the different vials with STIR and SPAIR are given in the Table. The T1 values (3T) are from the manufacturer model documentation. The simulated ADC values for composite vials were calculated from simulated signals using the individual vials T1 and ADC values, each vial contributing equally to the mixed signals (e.g., 50% fraction each for PVP10 and PVP40 for the PVP10/40 mix, 25% each for PVP10, PVP25 and 50% for PVP40 for the PVP10/25/2 × 40 mix). Right, top: The overestimation of the ADC values (mm²/s) with STIR appears more pronounced with TI = 300 ms (optimal value at 3T) than with TI = 200 ms. The overestimation is also more pronounced when the difference in the PVP T1s is larger (e.g., PVP14% + 40% compared to PVP25% + 40%)

While qualitative DWI might be acceptable for screening and lesion detection [16, 17], it has some limitations. For instance, benign lesions, such as cysts, with long T1 and T2 might appear very bright on DWI images, even at high *b-*values (so-called “shine-true” effect). Only the calculation of the ADC enables a clearer diagnosis, eliminating T1 and T2 effects, and revealing the underlying high ADC of such lesions. Regarding lesion classification or more advanced DWI applications, such as treatment monitoring, it is clear that quantitative DWI is mandatory, at least in the form of ADC values. A pitfall with STIR, though, is that ADC values obtained using the STIR “fat suppression” method are potentially biased toward the tissue components with the longest T1, as those components present in the lesion have higher signals at all *b*-values. This means that ADC values could become over- or most often underestimated, compared to the ground truth ADC (Fig. 3 right). While homogeneous lesions (with only one T1 component) will exhibit correct ADC values, the ADC of heterogeneous lesions (or regions of interest) with distributed T1 values becomes T1 weighted. This is shown in the phantom (Fig. 4): While the ADC values obtained in the homogeneous vials are similar for STIR and SPAIR, the ADC values obtained when mixing signals from vials with different T1 values significantly deviate from the SPAIR ADC values. In the phantom STIR ADC values are overestimated, as T1 and ADC values are correlated in the vials (i.e., long T1 values are associated with higher ADCs).

**Fig. 5:**
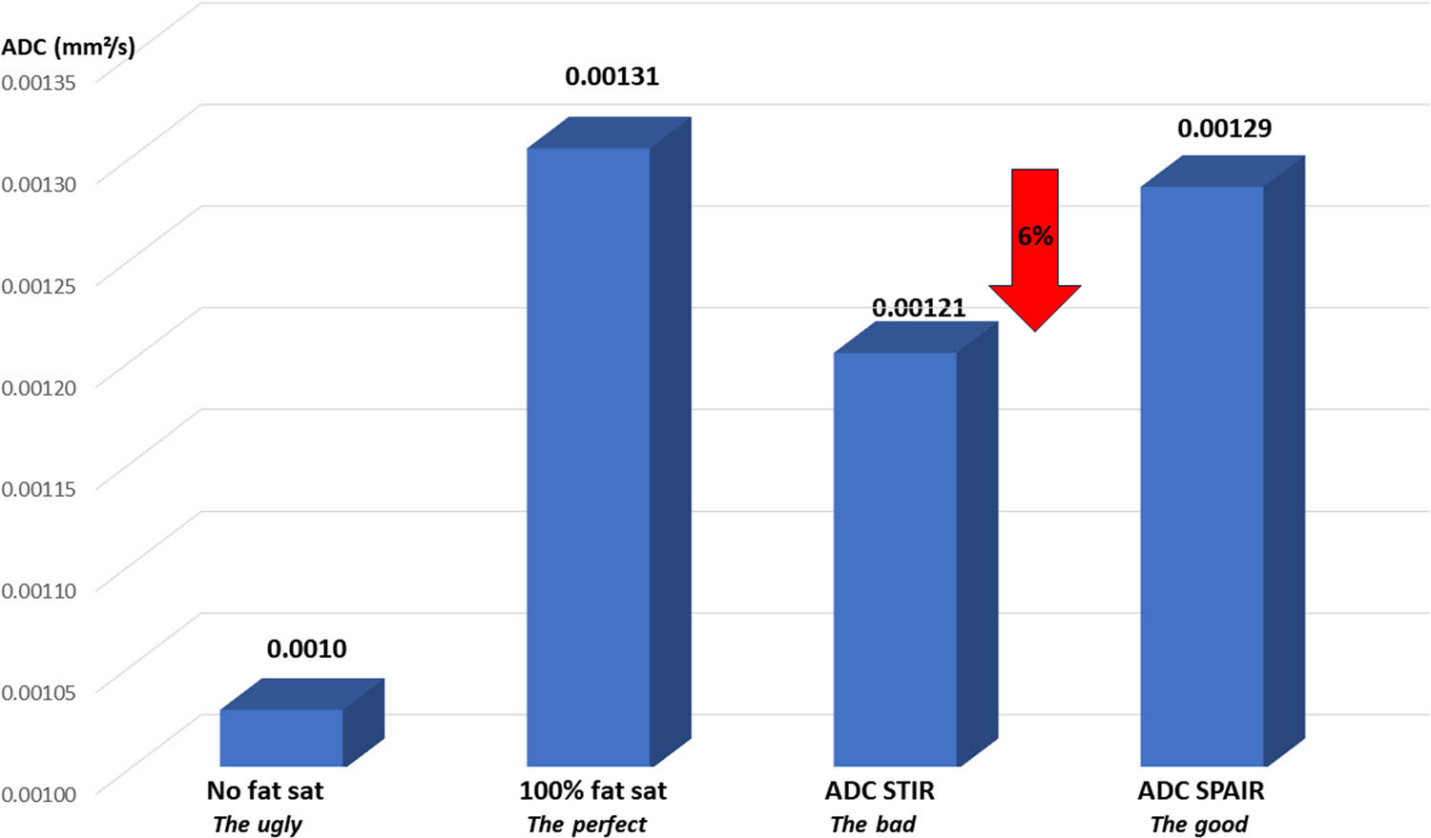
ADC values obtained in a simulated lesion according to the fat-signal suppression method. While the ADC is clearly very low when no fat suppression is applied (“the Ugly”) compared to the ADC value obtained in the absence of fat (“the perfect”), the ADC values obtained with the STIR method (“the Bad”) is lower than the reference ADC. The SPAIR ADC (“the Good”) remains closer to the ground truth in this example where the SPAIR fat-suppression level was assumed to be around 90%. The parameters used for the simulation are given in the Table in ESM Fig. 3

**Fig. 6:**
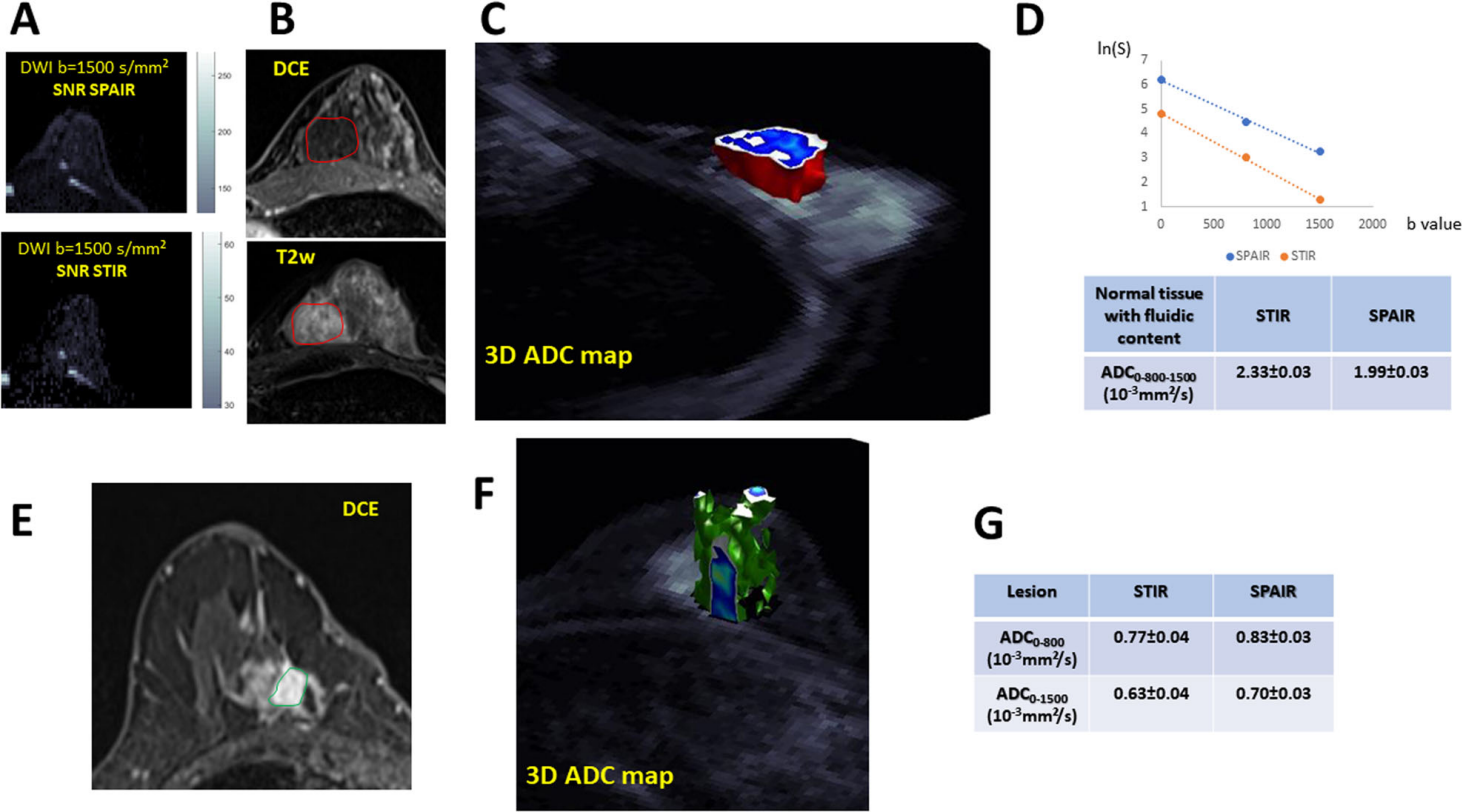
Clinical example cases. **A**, **B**, **C**, **D**: normal tissue with a fluidic component. **A**: SNR appears much lower for STIR than for SPAIR; **B**: DCE and T2w slices showing the Region-of-Interest; **C**: ADC map of the ROI (3D rendering); **D**: Plot of the ROI signal showing the lower STIR signal and Table with the ADC values (fit with *b* = 0, 800 and 1500 s/mm²). The STIR ADC is overestimated, as expected with a tissue comprising a fluidic component with long T1 and high diffusion. **E**, **F**, **G**: invasive ductal carcinoma **E**: DCE slice showing the region-of-interest; **F**: ADC map of the lesion (3D rendering); **G**: table with the ADC values (0–800 and 0–1500 s/mm²). The STIR ADC is underestimated, as expected with a tissue comprising a malignant component with long T1 and low diffusion

In malignant lesions, which usually have long T1 and low ADC values, the overall effect would be opposite, with an underestimation of ADC values with STIR, as shown in the simulations (Fig. 5) and with the invasive ductal carcinoma case (Fig. 6). This is a critical point for lesion classification based on ADC thresholds, as STIR ADC values might be underestimated.

## Discussion

### The Ugly

Because the diffusion coefficient of fat hydrogen nuclei is much lower than that of water, voxels or ROIs containing a mix of glandular or lesional tissues with fat exhibit artifactually lower ADC values than in the absence of fat. This problem is particularly important for the breast, compared to other tissues. This ADC underestimation depends on the amount and the nature of the fat content. Besides chemical shift artifacts which cause blurring and reduce lesion conspicuity, this pitfall becomes an important issue when considering quantitative DWI where ADC thresholds are used for lesion classification (e.g., benign versus malignant, where low ADC is an indicator of malignancy), Hence, it is of the utmost importance to suppress signals originating from fat in breast DWI. Here, we have investigated the two most popular methods used for fat suppression in breast DWI, highlighting the issues arising when using STIR, while the SPAIR method appears as the method of choice.

### The Bad

STIR relies on the use of a short inversion time (TI) designed to null out the signal from fat which has a short T1 value (optimal TI value is defined as T1_fat_.ln(2), hence for instance TI = 200 ms for T1_fat_ = 280 ms at 1.5 T and TI = 300 ms for T1_fat_ = 430 ms at 3 T). While TI is generally set once for a given acquisition protocol, the exact T1_fat_ value may vary across patients and across tissues, hence, the value chosen for TI remains approximative. Furthermore, the T1_fat_ value increases with the magnet operating field strength, B0, so TI will need to be adjusted between 1.5 T and 3 T. Also, as the inversion requires a well-defined 180° radiofrequency (RF) pulse, nulling out the fat signal assumes that the RF amplitude is fully controlled, hence that the RF (B1) is homogeneous, which might not be always the case depending on the geometry and design of the RF coils, especially in high-field MRI settings.

However, the most serious pitfall that is the method is not at all specific to fat: all tissue components with a T1 value close to that of fat will be erased from the DWI signal, as we have demonstrated. This situation is particularly detrimental when acquiring STIR/DWI data after the administration of a gadolinium-based contrast agent due to a sharp T1 decrease associated with contrast enhancement, as this can not only obscure tissues exhibiting contrast enhancement and decrease lesion visibility (similar to PVP40 in Fig. 2) but also result in a large ADC overestimation, thus mimicking benign lesions.

Another “hidden effect” comes from the effect of the noise on ADC values found within ROIs. The ROI ADC value is often obtained as the average of the ADC values for individual voxels within the ROI, taken from scanner-generated ADC maps. But it is not the same to average ADCs from ROI voxels using ADC maps as to calculate ADC values by first averaging the DWI signal intensities (e.g., from *b* = 0 and *b* = 800 s/mm² images, respectively) over the ROI voxels and then calculating the ADC from those averaged signals [1]. In the presence of noise, the ADC value averaged over the ROI voxels on the ADC maps is overestimated compared to the true ADC value. This difference is a consequence of the famous Jensen’s inequality which states that the average of a function (here ADC) of the signals is larger than the function of the averaged signals if the function is not linear (which is the case for the ADC) [1]. As STIR signals are more noisy than SPAIR signals, the ADC overestimation is potentially larger for STIR than SPAIR. This difference, however, can be mitigated by calculating ADC values from ROI-averaged raw signals.

Finally, let us mention another practical drawback of STIR, as the inversion pulse will have to be repeated many times when a large number of slices are acquired, leading to an increased RF absorption rate [18] and potential tissue heating, especially at high-field strength.

### The Good

While keeping the concept of the inversion recovery sequence to null out fat signals, effects of RF inhomogeneities can be eliminated using adiabatic RF pulses, where those RF pulses can be made spectroscopically specific to fat, hence leaving water DWI signals untouched [19]. This is the principle of the spectral adiabatic inversion recovery (SPAIR) method. With SPAIR, ADC values are not contaminated by the T1 profile within lesions and the overall SNR remains intact, leading to a better depiction of lesions (Figs. 2, 5, and 6).

One notable advantage of STIR is its versatility, as it can be effectively utilized across various field strengths and under diverse homogeneity conditions. Despite its versatility and effectiveness, it remains a less common choice compared to SPAIR in clinical practice. Studies have shown that DWI-SPAIR has a higher SNR and contrast-to-noise ratio (CNR) than DWI-STIR [2]. Noguiera et al compared SPAIR and STIR in breast lesion classification and showed that DWI-SPAIR provided higher sensitivity in lesion discrimination than STIR (85.1% versus 79.7%). SNR and CNR were significantly higher in DWI-SPAIR, while fat-suppression uniformity was superior in DWI-STIR [3].

This does not mean, however, that the SPAIR method does not have drawbacks either. As the fat-selective RF pulses must be tuned to the frequency of fat, any variations in the local magnetic field, B0, may lead to imperfect fat suppression, and, hence, to a possible underestimation of ADC values in tissues containing residual fat signals: The SPAIR method requires good B0 shimming. An alternative approach to mitigate the influence of the fat signal involves leveraging the relative phase difference between fat and water protons, a factor unaffected by B0 and B1 field inhomogeneity. This concept has found application in both water excitation and Dixon fat/water separation methodologies.

In recent developments, a combination of water excitation and spectral fat saturation which is independent of B0 and B1 field inhomogeneity has been proposed for breast imaging, offering a good CNR, albeit at the cost of slightly reduced SNR compared to SPAIR [20]. Water excitation is achieved through a composite RF pulse that encompasses an excitation pulse for both fat and water magnetization, followed by a delay that places fat magnetization 180 degrees out of phase with water magnetization. Subsequently, another identical excitation pulse is applied to align the fat magnetization with the longitudinal axis while further tilting the water magnetization toward the transverse plane. These investigations show promise and warrant further exploration to achieve optimal fat suppression in breast imaging.

## Conclusion

Effective fat-signal suppression is mandatory for qualitative and quantitative breast DWI. Among popular methods, SPAIR is preferable over STIR for fat-signal suppression in breast DWI, as STIR suffers from a loss in SNR, biased DWI contrast, and misestimation of ADC values biased towards tissue components with long T1 values. Accordingly, SPAIR has been recommended by EUSOBI [7], QIBA/RSNA (QIBA) [21], and the Korean Radiological Society [22].

## Supplementary information

ELECTRONIC SUPPLEMENTARY MATERIAL

## Abbreviations

ADC: Absolute diffusion coefficient
CNR: Contrast-to-noise ratio
DWI: Diffusion-weighted imaging
EPI: Echoplanar imaging
EUSOBI: European Society of Breast Imaging
FGT: Fibroglandular tissue
MRI: Magnetic resonance imaging
PVP: Polyvinylpyrrolidone
RF: Radiofrequency
SNR: Signal-to-noise ratio
SPAIR: Spectral adiabatic (or attenuated) inversion recovery
STIR: Short-tau inversion recovery
