# Supplementary material for: Fat-signal suppression in breast diffusion-weighted imaging: the Good, the Bad, and the Ugly
Source: Eur Radiol. 2024 Aug 7;35(2):733–41. doi: 10.1007/s00330-024-10973-4 (PMC11782347; doi:10.1007/s00330-024-10973-4)
Supplement: Supplementary file 1 — ELECTRONIC SUPPLEMENTARY MATERIAL [file 330_2024_10973_MOESM1_ESM.pdf]

Fat signal suppression in breast diffusion-weighted imaging: The Good, the Bad and the Ugly

ELECTRONIC SUPPLEMENTARY MATERIAL

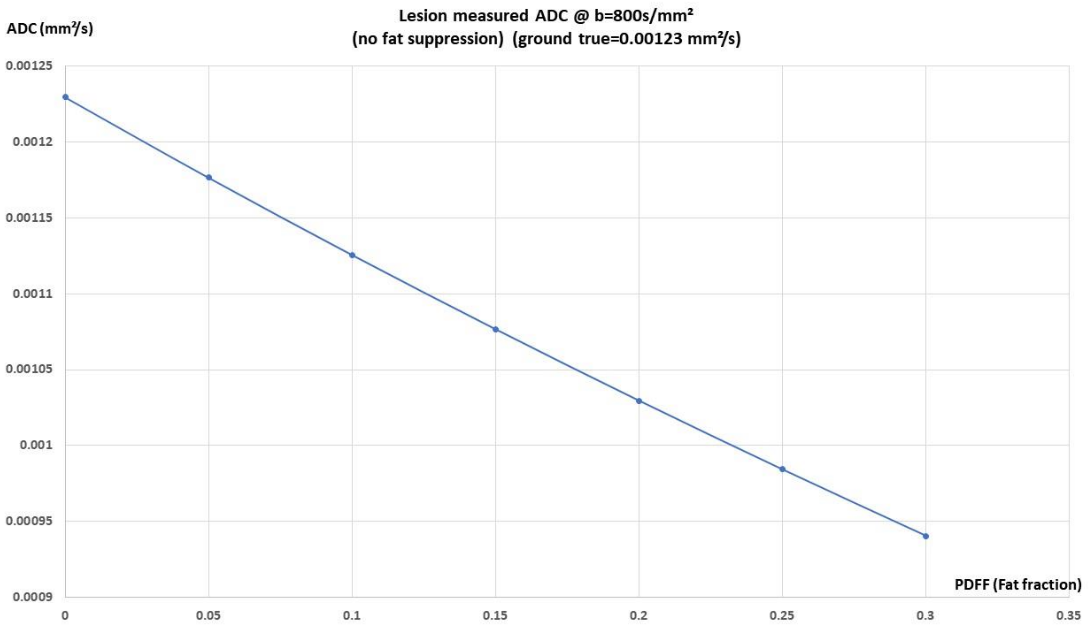

ESM Figure 1: Effect of fat on lesion ADC

Because of the very low diffusion coefficient of fat, the ADC obtained in a fat-containing lesion is reduced. Simulation performed using ADC values of 1.13 and 0.3 10^{-3}mm²/s for the lesion water and fat, respectively.

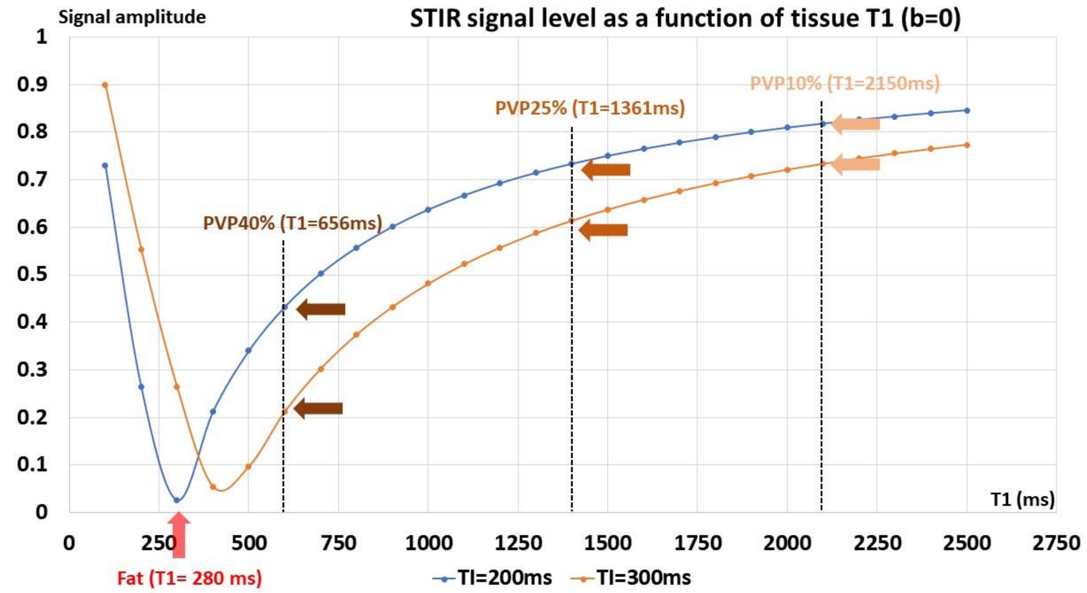

ESM Figure 2: Remaining signal after the 180° RF inversion pulse of the STIR method

When using the optimal TI value (here 200ms and 300ms for $T1_{fat}$*= 280ms (1.5T) and 430ms (3T), respectively) the fat signal is almost entirely nulled out. However, this effect is not fat specific: all tissues exhibit a signal loss, more for tissues with short T1 values, less for tissues with long T1 values, as shown here with stimulations for the PVP vials of the phantom.*

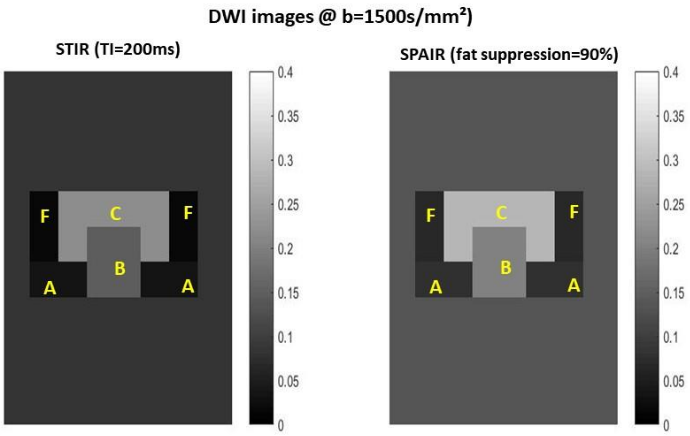

LESION	FAT	Tissue A	Tissue B	Tissue C
fraction (%)	0.2	0.3	0.3	0.2
T1 (ms)	280	500	1000	1700
D (mm²/s)	0.0003	0.0019	0.0014	0.0011
K	0	0.42	0.64	0.84

ESM Figure 3: STIR/DWI (TI=200ms, b=1500s/mm²) and SPAIR signal levels in a simulated lesion with various tissue components.

Overall, the signal level is lower with STIR, as all component signals are reduced. However, the contrast between the most malignant part of the lesion (C) and the background (B) is higher with STIR, because both the lesion low ADC and high T1 both contribute to contrast while only the ADC contributes with SPAIR. Parameters used for the simulation are given in the Table.
